# Protein and Glycan Mimicry in HIV Vaccine Design

**DOI:** 10.1016/j.jmb.2019.04.016

**Published:** 2019-05-31

**Authors:** Gemma E. Seabright, Katie J. Doores, Dennis R. Burton, Max Crispin

**Affiliations:** 1 Oxford Glycobiology Institute, Department of Biochemistry, University of Oxford, Oxford, OX1 3QU, UK; 2 School of Biological Sciences, University of Southampton, Southampton, SO17 1BJ, UK; 3 Department of Infectious Diseases, King's College London, Guy's Hospital, London, SE1 9RT, UK; 4 Department of Immunology and Microbiology, the Scripps Centre for HIV/AIDS Vaccine Immunology and Immunogen Discovery (CHAVI-ID), International AIDS Vaccine Initiative Neutralizing Antibody Centre, Scripps Research, La Jolla, CA 92037, USA

**Keywords:** HIV-1, human immunodeficiency virus type 1, Env, envelope spike, AIDS, acquired immune deficiency syndrome, bnAb(s), broadly neutralizing antibody(ies), nAb(s), neutralizing antibody(ies), gl-bnAb(s), germline-bnAb(s), CD4bs, CD4 binding site, CCR5, C-C chemokine receptor type 5, CXCR4, C-X-C chemokine receptor type 4, TF, transmitted/founder, EM, electron microscopy, PNGS, potential *N*-glycosylation sites, BCR, B cell receptor, IMP, intrinsic mannose patch, TAMP, trimer-associated mannose patch, HCDR3, third heavy-chain complementarity-determining regions, CDR, complementarity-determining regions, LOS, lipooligosaccharides, SP, signal peptide, MPER, membrane proximal external region, TM, transmembrane region, CT, cytoplasmic tail, HR1/2, heptad repeat 1 or 2, NFL, native flexibly linked, SC, single-chain, UFO, uncleaved prefusion-optimized, PBMC, peripheral blood mononuclear cell, PNS, peripheral nervous system, CHO, Chinese hamster ovary, HEK, human embryonic kidney, cGMP, current good manufacturing practices, Glc, glucose, Man, mannose, GlcNAc, *N*-acetylglucosamine, Gal, galactose, Fuc, fucose, Neu5Ac, *N*-acetylneuraminic acid (sialic acid), GlcN, glucosamine, KDO, 2-keto-3-deoxy-D-manno-octulosonic acid, ER, endoplasmic reticulum, α-man I and II, α-mannosidase I and II, GnT I, *N*-acetylglucosaminyltransferase I, human immunodeficiency virus, vaccinology, antibodies, glycosylation, structure

## Abstract

Antigenic mimicry is a fundamental tenet of structure-based vaccinology. Vaccine strategies for the human immunodeficiency virus type 1 (HIV-1) focus on the mimicry of its envelope spike (Env) due to its exposed location on the viral membrane and role in mediating infection. However, the virus has evolved to minimize the immunogenicity of conserved epitopes on the envelope spike. This principle is starkly illustrated by the presence of an extensive array of host-derived glycans, which act to shield the underlying protein from antibody recognition. Despite these hurdles, a subset of HIV-infected individuals eventually develop broadly neutralizing antibodies that recognize these virally presented glycans. Effective HIV-1 immunogens are therefore likely to involve some degree of mimicry of both the protein and glycan components of Env. As such, considerable efforts have been made to characterize the structure of the envelope spike and its glycan shield. This review summarizes the recent progress made in this field, with an emphasis on our growing understanding of the factors shaping the glycan shield of Env derived from both virus and soluble immunogens. We argue that recombinant mimics of the envelope spike are currently capable of capturing many features of the native viral glycan shield. Finally, we explore strategies through which the immunogenicity of Env glycans may be enhanced in the development of future immunogens.

## Highlights

• A focus of HIV-1 vaccine design is the development of soluble, recombinant envelope spike mimics.
• The envelope spike glycan shield acts to protect the underlying protein from immune recognition but can be targeted by broadly neutralizing antibodies.
• An understanding of the factors shaping viral and immunogen glycosylation will help guide the design of immunogens.
• Protein structural mimicry of HIV immunogens helps drive mimicry of the dense glycan coat.
• Strategies that increase the immunogenicity of glycan-dependent epitopes are likely to be required.

## Graphical Abstract

**Figure:**
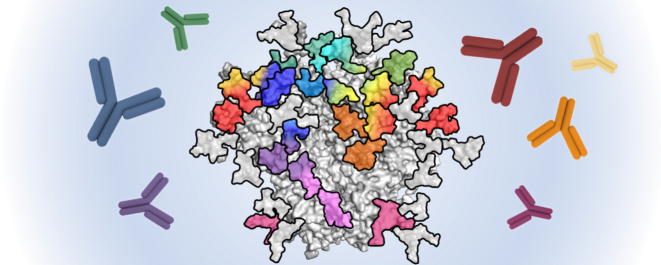

## Challenges Facing HIV-1 Vaccine Design

Vaccines typically contain or mimic parts or all of a pathogen, such as an attenuated strain or recombinant soluble surface protein, to prime the immune system to produce an effective response upon future exposure to that pathogen. This strategy has proved to be very successful in the past, famously resulting in the complete eradication of the smallpox virus [1], and more recently in a protective vaccine against Ebola virus [2]. Despite significant efforts, a vaccine capable of eliciting a protective response against the human immunodeficiency virus type 1 (HIV-1) has proved elusive [3].

Both antibodies and cytotoxic T lymphocytes are produced upon infection with HIV-1. However, the virus has evolved several features that undermine immunological control and eradication of infection, most notably, very high antigenic diversity and the establishment of a latent viral reservoir. While treatment with antiretroviral drugs can extend the life expectancy of infected individuals to near-normal [4], [5], drug resistance has been documented for every class of antiretroviral currently in use [6], and treatment regimens are often accompanied by adverse effects and low levels of adherence. Furthermore, cessation of therapy results in rapid viral rebound [7]. If left untreated, HIV-1 infection results in diminished numbers of CD4 + T cells (the major viral target cell), causing acquired immune deficiency syndrome (AIDS) and death. While HIV-1 cure strategies are an important and viable field of research [8], the development of an effective prophylactic vaccine remains a primary goal in the effort to control the HIV-1 pandemic.

Analysis of the immune response of infected individuals has renewed optimism that a vaccine may be a tractable goal [9], [10]. A subset of HIV-1 infected patients are able to generate antibodies of sufficient breadth and potency to neutralize the vast majority of circulating HIV-1 isolates [11], [12], [13]. Although these broadly neutralizing antibodies (bnAbs) are unable to clear the virus from the infected individual, they are able to protect non-human primates [14], [15], [16], [17], [18], [19], [20], [21], [22], [23], [24], [25] and humanized mice [26], [27], [28], [29], [30], [31], [32] from viral challenge when passively administered. Importantly, these antibodies are protective at concentrations achievable by vaccination in other settings [15], [17]. Taken together, these observations provide some support for the hypothesis that a vaccine can be developed capable of generating a protective antibody response against HIV-1.

**Fig. 1:**
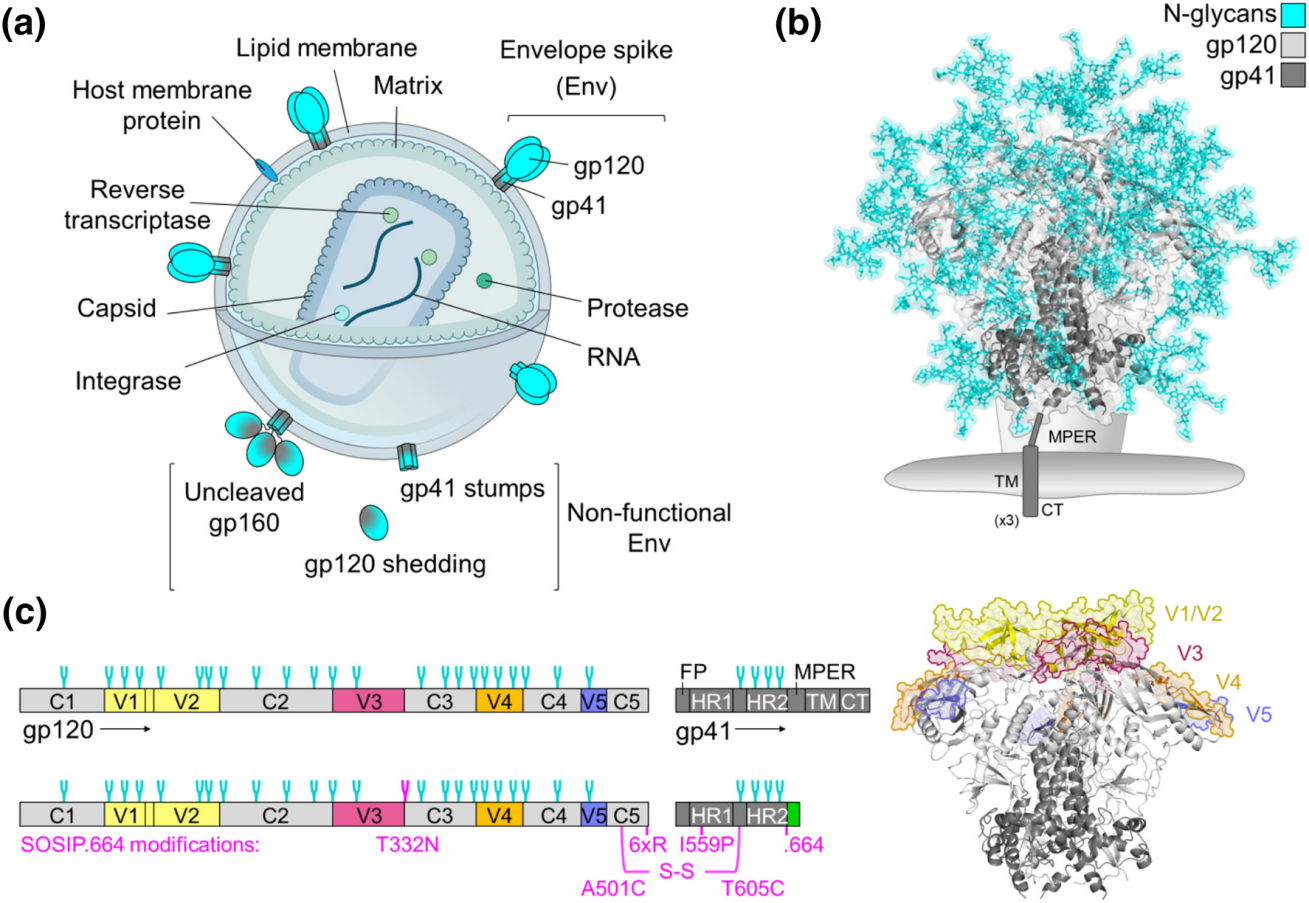
Structure of the HIV-1 virion and the envelope spike. (a) Graphic depicting the structure of the HIV-1 virion. Approximately 14 envelope spikes are displayed on the surface of the virion (mean for one HIV-1 isolate), embedded into the host cell-derived lipid membrane [36]. (b) Model of a fully glycosylated envelope spike (glycans in cyan sticks) based on PDB: 5ACO [37]. Glycans were added according to Behrens *et al*. [38]. The envelope spike is a trimer of non-covalently associated gp120 (light gray) and gp41 (dark gray) heterodimers. The gp120 subunits contain the CD4 receptor and CCR5 or CXCR4 co-receptor binding sites. Upon binding, the trimer undergoes substantial conformational changes that enable the gp41 subunits to drive fusion of the viral and host cell membranes. The membrane proximal external region (MPER), transmembrane domain (TM) and cytoplasmic tail (CT) are not present on the structure and are shown for one protomer in cartoon along with the lipid membrane for orientation. (c) Left: Schematic representation of the primary structure of Env (top) and the soluble immunogen, BG505 SOSIP.664 (bottom). Variable regions (V1–5) are shown in colour, constant regions (C1–5) are shown in light gray, and gp41 is shown in dark gray. The envelope spike has approximately 25 potential *N*-glycosylation sites per gp120, and 4 per gp41 (cyan forks; mean across many isolates) [39]. SOSIP.664 modifications are annotated in magenta, with optional purification tag coloured green. FP, fusion peptide; HR1/2, heptad repeat 1 and 2. Right: Model of a de-glycosylated envelope spike (as in panel b), with variable loops coloured accordingly.

All known bnAbs are directed against the envelope spike (Env) [33], [34], [35], the only viral protein on the virus surface (Fig. 1a). Therefore, while the contribution of T cells in the development of an antibody response is critical [40], [41], [42], considerable research efforts have been directed at the development of stable, recombinant mimics of the envelope spike in order to elicit a B cell response [43]. Central to this strategy is the hypothesis that antigenic mimicry of a vaccine candidate is essential for the induction of an antibody response against that antigen [41], [44]. However, HIV-1 has evolved under immense selection pressure by the humoral immune system, and consequently many of the most valuable bnAb epitopes are inherently poor immunogens. One manifestation of this is an extensive array of host-derived *N*-glycans which surrounds the envelope spike to create a largely immunologically “self” glycan shield (Fig. 1b). While originally thought to protect the underlying protein surface from immune recognition, the discovery that many bnAbs can develop that recognize glycan epitopes has exposed the glycan shield itself as a potential target for vaccine design [45]. Scanlan *et al*. [46] previously highlighted the apparent contradiction in that HIV-1 glycans have evolved as an adaptation for virus survival and yet have emerged as targets for vaccine design. It is therefore important that Env-based immunogens are able to mimic effectively both the protein and glycan components of the envelope spike, although strategies that tackle the poor immunogenicity of the glycan epitopes are likely to be required.

In order to inform the rational design of Env immunogens, a detailed understanding of both the composition of the glycan shield and the structural rules governing its formation are required. This review will discuss recent contributions to the field of HIV-1 vaccine design, specifically the principles governing HIV-1 glycosylation and how this can be used to help select candidate immunogens. We also discuss the strategies being explored with the aim of boosting the immunogenicity of Env-based vaccines.

### The antibody response

Neutralizing antibodies (nAbs) typically work by binding an antigen on the viral surface to prevent the virus from infecting the host cell, and correlate with protection in the majority of licensed vaccines [47]. As the only viral protein on the virion surface and playing a key role in mediating infection, the envelope spike is the sole target for nAbs. However, several fundamental features of HIV-1 biology hinder the development of nAbs in both a vaccine and infection setting. The functional envelope spike is a trimer of non-covalently associated gp120–gp41 heterodimers (Fig. 1), generated by furin cleavage of a gp160 precursor polypeptide. Host cell tropism and attachment is mediated by the gp120 subunits, which contain the CD4 receptor and co-receptor (CCR5 or CXCR4) binding sites. Once bound, substantial conformational changes enable the gp41 subunits to drive fusion of the viral and host cell membranes [48]. Antibodies capable of binding the spike in its functional conformation prevent this occurring. However, functional Envs are few and far between, with only approximately 14 spikes per viral particle (mean for one HIV-1 isolate) [36]. The wide spacing of the envelope spikes is disadvantageous for the host antibody response as B cells are more effectively activated by repetitive and organized structures [49].

The virus also produces an abundance of non-functional envelope spikes in the form of uncleaved gp160, and non-functional monomeric subunits such as soluble gp120 and gp41 stumps. These can arise through either the improper processing of the spike or its later disintegration (Fig. 1a). This “viral debris” displays immunodominant epitopes that are either occluded or absent on the functional trimer (e.g., the inner surface of gp120), and acts to divert the host antibody response [50], [51], [52], [53], [54], [55]. Thus, the initial antibody response, arising over the first few weeks of infection, is incapable of binding the functional envelope spike and is “non-neutralizing” [56].

A further nAb evasion feature of HIV-1 is the relatively poor accessibility of its conserved epitopes. The CD4 binding site (CD4bs), for example, is a highly conserved region essential for infectivity and thus represents a potentially vulnerable site for antibody neutralization. However, its recessed location within the trimer interface, surrounded by *N*-glycans, reduces the accessibility of this valuable collection of epitopes for nAb recognition. Interestingly, llamas and cows are capable of generating nAbs against the CD4bs region following immunization with Env [57], [58]. Llamas naturally produce heavy-chain only antibodies that are much smaller than conventional antibodies, while cow antibodies contain very long third heavy-chain complementarity-determining regions (HCDR3), some over 70 amino acids in length. The unusual architectures of these antibodies enable them to easily access the CD4bs, supporting a model of steric blocking for conventional IgG molecules.

In contrast, highly variable regions occupy the more accessible regions of the trimer, providing yet another immunodominant diversion (Fig. 1c). Within a few months of infection, patients readily develop autologous nAbs (i.e., antibodies capable of neutralizing only the strain they were raised against), often directed at the variable loops 1, 2 and 3 (V1/V2 and V3) [59], [60]. Antibody-mediated selection pressure, combined with an error-prone viral reverse transcriptase, rapidly drives viral escape and results in extreme diversity [60]. Indeed, the genetic diversity of HIV-1 within an infected individual is comparable to the global genetic diversity of influenza in one year [39]. Thus, an effective vaccine against HIV-1 must induce not only nAbs, but also antibodies with sufficient breadth of activity to neutralize the majority of circulating strains (bnAbs).

The development of bnAbs is dependent on the activation of the appropriate naïve B cells by engaging their B cell receptor (BCR), that is, the precursor bnAb, prior to affinity maturation of the B cell in the germinal center by somatic hypermutation. The resulting bnAbs are often significantly mutated from their germline-encoded BCR [33]. However, this process is hindered by the low affinity of so-called “germline”-bnAbs (gl-bnAbs) to the envelope spike. Consequently, many recombinant Env mimics also fail to bind gl-bnAbs [61], [62], [63], [64], adding to the difficulties in eliciting bnAbs in a vaccine setting (Mimicking bnAb development in natural infection section).

### The glycan shield

A contributing factor to the immunodominance of many of the non-neutralizing and autologous neutralizing epitopes, and the inability of Env to bind gl-bnAbs, is the presence of an array of *N*-glycans that mask much of the surface of the envelope spike (Fig. 1b). Each Env can have upwards of 90 potential *N*-glycosylation sites (PNGS), with glycans comprising approximately half the trimer's mass [65]. The extensive *N*-glycosylation presents additional challenges for the host antibody response to overcome. The glycans are derived from the host's own glycosylation machinery during Env synthesis and are therefore considered immunologically “self.” Auto-reactive B cells undergo strong negative selection during B cell development, constraining the development of potential anti-glycan antibodies [66]. Furthermore, glycoproteins tend to exist as heterogeneous populations, with a multitude of glycan structures decorating the same protein backbone, thereby potentially reducing the antigenicity of each individual glycoform [67]. Lastly, protein–glycan interactions tend to have low binding affinities and often require multivalent interactions to overcome this [68]. The heavily glycosylated outer domain of gp120 has been dubbed the “silent face” of HIV-1, due to the previous lack of antibodies described against this region [69].

The role of glycans in protecting HIV-1 from neutralizing antibody responses has been well documented. The glycan shield constantly evolves to escape the host immune system, with the addition and deletion of glycan sites frequently used by the virus to escape nAb responses [70], [71], [72]. The “evolving glycan shield” escape response is typified by the N332 glycan, which has been reported to shift from the N334 position and back again after the appearance of N332-dependent nAbs [70]. Furthermore, transmitted/founder (TF) viruses typically have fewer PNGS than chronic isolates [72], [73], [74], [75]. While this suggests that there may be a fitness advantage to having fewer PNGS, a balance must then be struck between maintaining viral fitness and protecting vulnerable epitopes from the emerging nAb response. More recently, a study by Wagh *et al*. [76] reported that the addition of PNGS to fill holes in the glycan shield *in vivo* resulted in increased resistance to autologous nAbs. Indeed, many *in vitro* studies have also reported the increased susceptibility of the virus to neutralization upon removal of PNGS [77], [78], [79], [80], [81], which can often be rationalized by clashes observed between nAbs and glycans in structural studies [82], [83].

Despite the above-mentioned challenges, approximately a third of infected individuals develop some level of bnAbs after a few years of infection [11], [12], [13]. Many bnAbs are able to either penetrate the glycan shield to bind protein surfaces or directly bind to Env glycans. Thus, while glycan shielding remains a potent immune evasion strategy, the discovery of numerous glycan-binding bn has highlighted the glycan shield as part of an attractive target for vaccine design [84]. The importance of the glycan shield in HIV-1 vaccine design has recently been underlined by Wagh *et al*. [76], who observed that the development of bnAbs in infected individuals correlated with the completeness of the glycan shield at transmission.

### Instability of the viral spike

Although bnAbs are increasingly being isolated from infected individuals, we are yet to elicit them in a vaccine setting in humans. Early vaccination strategies using recombinant, monomeric gp120 failed to confer protection [85], [86], [87], presumably due to the elicitation of antibodies directed against the aforementioned immunodominant non-neutralizing epitopes (and/or the absence of gp41 and quaternary epitopes) [88]. The focus of HIV-1 vaccine research has now shifted to include the production of trimeric Env immunogens that display the majority of bnAb epitopes while minimizing non-neutralizing epitopes as much as possible, with the hope that these will be better able to induce a bnAb response [43]. However, the envelope spike is inherently unstable, reflecting its need to undergo substantial conformational changes during viral and host cell fusion. This has made the design of native-like immunogens particularly challenging (for an extensive review on the history and design of native-like Env trimers, see Sanders and Moore [43]).

The desire to remove the transmembrane region of the protein in order to generate soluble mimics often amplified trimer instability. Initial efforts to stabilize the trimer involved the removal or inactivation of the furin cleavage site to prevent gp120–gp41 dissociation, and/or the introduction of a trimerization domain at the C-terminus to prevent separation of the three gp120–gp41 heterodimers [89], [90]. Although these approaches usually generated trimers, there was often an abundance of monomers, dimers, and higher-molecular weight aggregates, owing to the inappropriate formation of intermolecular disulfide bonds [91], [92]. These trimer constructs often displayed aberrantly folded gp120s due to intramolecular disulfide bond scrambling and non-native like quaternary structures as judged by peptide mapping, negative-stain electron microscopy (EM), hydrogen–deuterium exchange mass spectrometry and glycosylation analysis (Protein structure and the trimer-associated mannose patch section). Perhaps most importantly, these trimers display non-native-like antigenicity [91], [92], [93], [94], [95] and are commonly referred to as “pseudotrimers” to reflect their various non-native properties [43]. With hindsight, it was therefore unsurprising that immunization studies with pseudotrimers, not unlike monomeric gp120, failed to elicit nAbs with sufficient breadth or potency [96], [97], [98].

## Protein Mimicry in Vaccine Design

The development of the SOSIP.664 platform transformed the field of native-like HIV-1 trimer design (Fig. 1c) [43]. These constructs retained the furin cleavage site, optimized for efficient cleavage and relied on the introduction of a disulfide bond (“SOS”) to covalently link the gp120 and gp41 subunits [99]. An additional point mutation in the gp41 subunits (I559P, “IP”) strengthened interactions between the three heterodimers by trapping the trimer in its pre-fusion conformation [100], [101], while truncation before the transmembrane region (“.664”) ensured the solubility of the trimers and reduced aggregate formation [102]. A further point mutation (T332N) introduced a glycan site that contributes to the epitope of many bnAbs. This format was first successfully applied to a clade A strain, BG505 [54], with the resulting “BG505 SOSIP.664” trimers exhibiting both native-like structure [103] and antigenicity [54].

In recent years, an arsenal of native-like trimers has been produced. The “SOSIP” format has since been applied to multiple strains, and mosaic and consensus sequences, although these often required further stabilizing point mutations and disulfide bonds (either between gp120–gp41 and/or between protomers) [104], [105], [106], [107], [108], [109], [110], [111], [112]. Other native-like trimer formats focused on eliminating the requirement for furin cleavage (and therefore the need to co-express Env immunogens with furin encoding plasmids) in an attempt to simplify protein production strategies and DNA-based vaccines. Native flexibly linked (NFL) and single-chain (SC) trimers achieved this feat by replacing the furin cleavage site with a flexible Gly–Ser linker [113], [114]. While the NFL and SC constructs both relied on the I559P point mutation to maintain the pre-fusion conformation, UFO (uncleaved prefusion-optimized) constructs achieved this through a computational redesign of the HR1 (heptad repeat 1) region [115]. The immunogenicity of many of the above-mentioned native-like trimers has now been investigated in animal models [104], [107], [116], [117], [118], [119], [120], [121] and has been reviewed by Sanders and Moore [43].

## Display of bnAb Epitopes by Viral Spike Mimetics

**Fig. 2:**
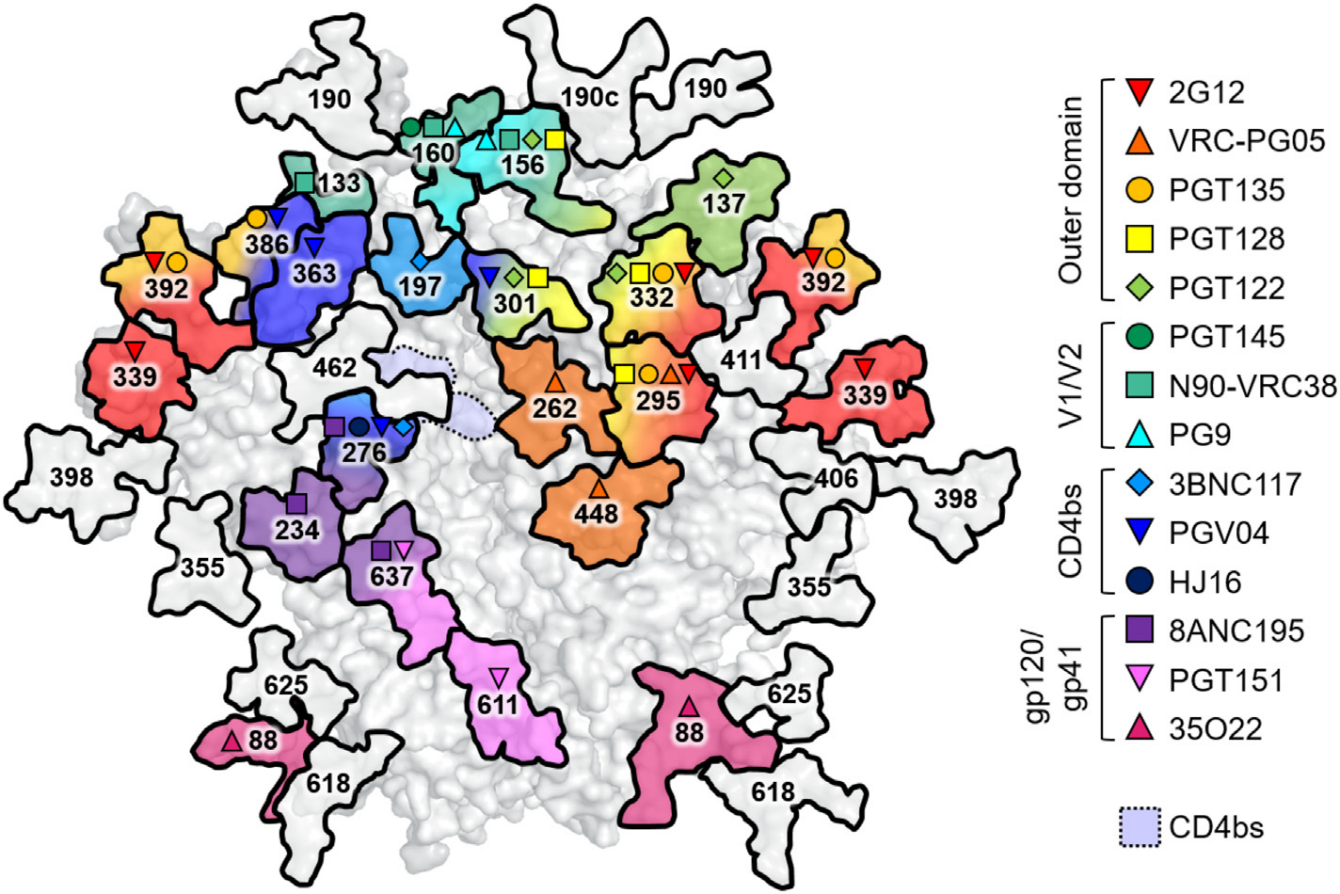
Broadly neutralizing antibodies recognize protein‐glycan epitopes. Model of a fully glycosylated BG505 SOSIP.664 trimer depicting the *N*-glycans that have been implicated in binding a variety of bnAbs (inset key). Glycans not present on the BG505 strain have been omitted; for example, 35O22 also recognizes *N*-glycans at positions 230 and 241. Model based on PDB: 5ACO as in Fig. 1, numbering according to the HXB2 reference sequence. CD4bs, CD4 binding site.

Native-like trimers are often assessed by their ability to bind bnAbs and not non-neutralizing antibodies. The development of native-like soluble trimers enabled the characterization of many bnAb epitopes through various biophysical techniques, such as x-ray crystallography and EM [122]. Broadly neutralizing antibodies are generally categorized by their recognition of five distinct and largely conserved epitopes on the envelope spike: the CD4 binding site (CD4bs), the membrane proximal external region (MPER), and the *N*-glycans located at the gp120–gp41 interface, the outer domain of gp120, and on the V1/V2 loops at the trimer apex. However, recent advances in high-throughput B cell screening have led to a dramatic increase in the identification of new bnAbs and have subsequently revealed a continuum of epitopes spanning the entire surface of the trimer [34], including the majority of *N*-glycans (Fig. 2). The recent characterization of bnAb VRC-PG05, which recognizes glycans at positions N262, N295 and N448, could conceivably be the last class of bnAb to be identified as its discovery filled one of the few remaining gaps on the trimer surface [123].

The extent to which individual bnAbs depend on glycans for binding and neutralization varies. At one extreme, 2G12 recognizes an epitope composed exclusively of *N*-glycans [124], [125], [126], [127], [128], [129], [130]. On the other hand, bnAbs against the CD4bs, for example, have evolved to either avoid or accommodate glycans that would otherwise occlude the underlying protein epitope [131]. Most bnAb epitopes occupy a midway point, with binding dependent on both protein and glycan components.

The abundance of glycan-targeting bnAbs has earmarked *N*-glycans as important components of a future HIV-1 vaccine. Thus, there is a need for a detailed definition of the precise composition of the glycan shield on both viral Env and candidate immunogens. In addition, an understanding of the principles controlling glycosylation will help guide the design of immunogens that are able to effectively mimic viral Env glycosylation, and, hopefully, elicit a broadly neutralizing anti-glycan response.

### Structure and development of broadly neutralizing antibodies

The development of bnAbs requires repeated rounds of viral escape and antibody maturation and can therefore take several years of infection to arise [70], [132], [133]. Their slow development may reflect the fact that bnAbs often depend on unusual antibody features in order to overcome the aforementioned challenges associated with the development of glycan recognition and neutralization breadth.

The glycan-targeting antibody, 2G12, overcomes low-affinity protein‐glycan interactions by exhibiting a unique domain-exchanged structure. Here, two heavy-chain variable regions are exchanged to create a single Fab_2_ [127]. The resulting structure has an additional antigen binding site at the interface of the two arms, which allows for the binding of four Env glycans with high avidity [126], [127].

Many bnAbs, particularly those targeting the V1/V2 and outer domain glycans, contain very long HCDR3 sequences [134], [135], [136], [137], [138], [139]. While the average length of human HCDR3 is 13 residues long [140], bnAb HCDR3s can contain upwards of 30 residues. This is particularly true of bnAbs targeting the glycans of the outer domain and V1/V2 loops, where long HCDR3 sequences allow the antibody to penetrate the glycan shield and make contact with the underlying protein surface. The HCDR3 of PGT145, for example, contains 33 residues, and penetrates the N160 glycan triad at the apex of the trimer to contact underlying protein residues from all three protomers [134]. For outer domain-targeting bnAbs (e.g., PGT128 and PGT135), long HCDR3s allow penetration of the glycan shield to access the base of the V3 loop [136], [137]. In both examples, at least one glycan makes extensive interactions with the bnAb binding site. A similar phenomenon has been observed for bnAbs targeting the MPER, namely, 4E10, where a long (20 residue), hydrophobic HCDR3 allows for contact with the lipid membrane as well as gp41 protein surface [139]. Often long HCDR3s are accompanied by post-translational modifications, such as tyrosine sulfation, which have also been reported to contribute to binding and neutralization [135].

Another feature present in many bnAbs is extensive somatic hypermutation [33]. Classically, antibodies accumulate such mutations in the antigen-contacting complementarity-determining regions (CDRs). However, bnAbs often require somatic mutations to the more conserved framework regions [141]. Although there is evidence that higher levels of somatic hypermutation correlate with increased breadth and potency in some bnAb lineages [142], this may be a consequence of the length of time of development rather than a necessity of bnAb activity. For instance, partially germline reverted forms of VRC01 and 10E8 bnAbs are still broad and potent neutralizers [143].

Poly- and auto-reactivity are also frequently associated with bnAbs [144], [145], [146]. These observations are somewhat unusual as both characteristics are negatively selected for during B cell development [66]. It has been hypothesized that conserved HIV-1 epitopes may mimic host proteins in order to avoid host antibody responses through the down-regulation of relevant B cells by host tolerance mechanisms [144], [145], [147]. A similar host immune evasion strategy has been reported for the pathogen *Campylobacter jejuni*, whose lipooligosaccharide (LOS) mimics the gangliosides of the host's peripheral nervous system [148]. Despite these barriers, poly- and auto-reactive bnAbs are not uncommon, although their development may be dependent on prolonged exposure to the antigen, as several B cell tolerance checkpoints may need to be overcome. The elicitation of bnAbs through typical vaccination strategies may therefore prove challenging.

## Glycan Mimicry in Vaccine Design

The observation that glycan-dependent bnAbs can bind to soluble mimetics of the viral spike indicates that the glycans displayed by such immunogens are largely tolerated within the bnAb glycan specificity profile. However, given the nature of bnAbs, which have evolved to tolerate microheterogeneity of glycans, cross-reactivity does not necessarily indicate that the target glycans are precisely conserved between immunogen and virus. For example, some glycan-specific bnAbs recognize the largely invariant base of the glycan [134], [149]. Furthermore, failure of an immunogen to bind a bnAb could be symptomatic of either a failure of protein mimicry, glycan mimicry, or both. For these reasons, there is significant interest in defining the glycosylation of both target viruses and candidate immunogens. Finally, detailed information about glycosylation will also help define the immunological ramifications of particular immunogen and viral glycoforms that may go beyond simply the display of particular glycan structures at a particular site. For example, are some glycoforms more inflammatory or immunogenic than others [150]?

### Understanding Env glycosylation processing

The analysis of HIV-1 glycosylation is particularly challenging given the extensive heterogeneity displayed by glycoproteins, combined with the large number of PNGS on Env. Furthermore, isolating virally derived Env in sufficient quantities for glycan analysis has proved difficult. Early glycan analyses were therefore generally performed on recombinant, monomeric gp120 [151], [152], [153], [154], [155], [156], [157], or trimeric Env constructs derived from pseudovirions [151], [158], [159], [160], membrane-associated trimers [161], [162], and recombinant, soluble trimers [38], [94], [121], [162], [163], [164], [165], [166], [167], [168], [169], [170]. These analyses soon revealed key aspects of Env glycosylation processing. Firstly, the Env glycan shield is heterogeneous. The gp120 subunit alone can contain upward of 50 different glycan structures [38], attributed to the large number of glycan processing enzymes possessed by mammalian cells. Despite this heterogeneity, there always exists a substantial population of under-processed, oligomannose-type glycans (Man_5-9_GlcNAc_2_). This glycan signature arises through steric constraints within the Env glycan shield that impede the actions of some of the host glycosylation enzymes (Glycan clustering and the intrinsic mannose patch section and Protein structure and the trimer-associated mannose patch section) [156], [158], [159], [171].

Many of the above glycan analyses focused on the analysis of enzymatically released *N*-glycans. While this provides a useful readout of the overall glycosylation profile, no site-specific information can be gleaned. Such information, which involves the analysis of protease-digested glycopeptides, is valuable in order to elucidate the precise composition of bnAb epitopes. The development of the SOSIP.664 platform, combined with an advancement of mass spectrometric and chromatographic technologies enabled more in-depth, site-specific glycan analyses.

**Fig. 3:**
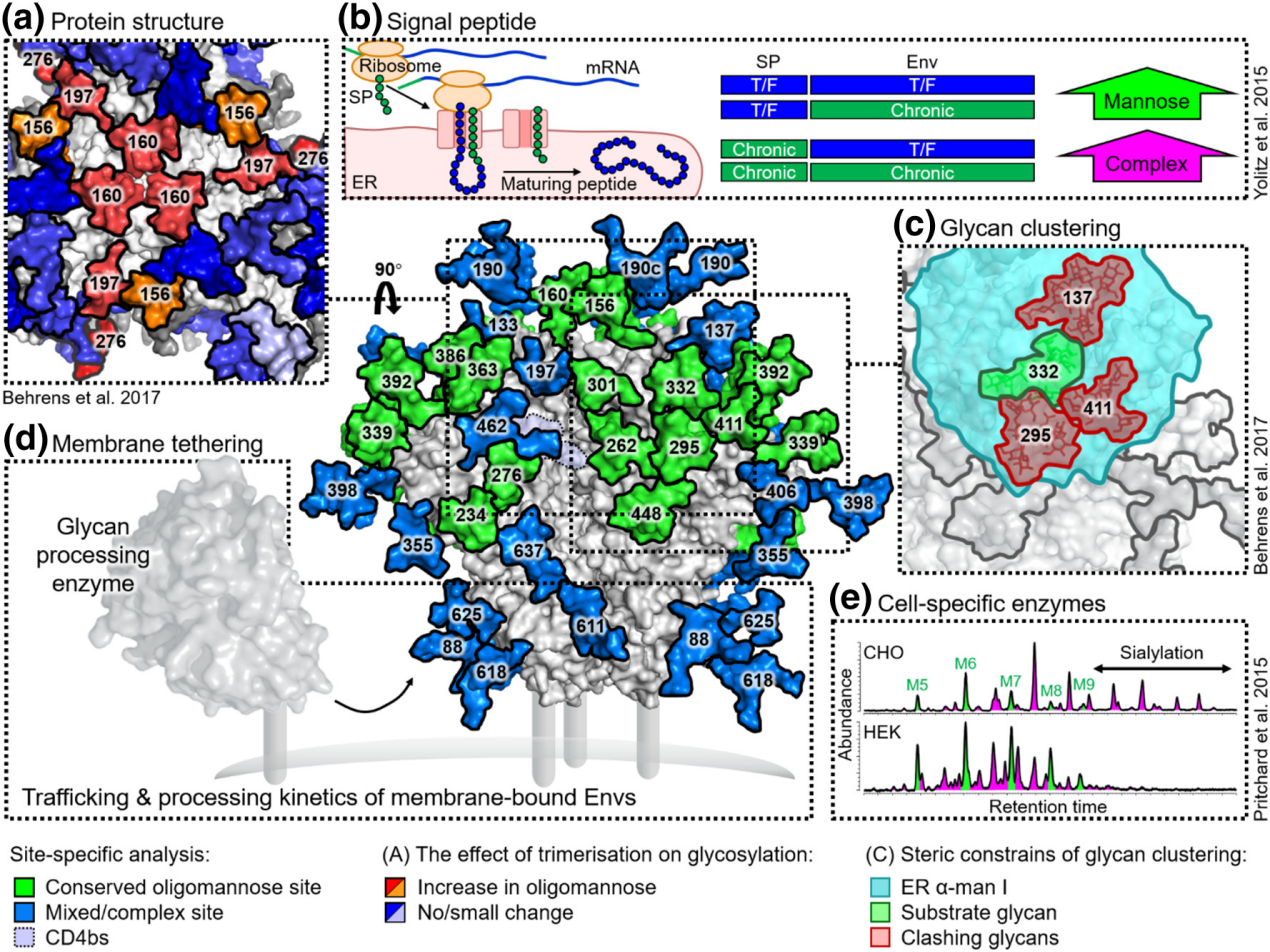
Principles controlling Env glycosylation. Site-specific glycan analysis of recombinant BG505 SOSIP.664 [38], [163], [168], [172] and virally derived BG505 Env [173], [174] has revealed clusters of glycans displaying under-processed, oligomannose-type glycans (green). These are largely located on the outer domain of gp120 (forming the intrinsic mannose patch) and at the trimer apex and protomer interfaces (forming the trimer-associated mannose patch). Model according to Fig. 1. (a) The quaternary protein structure of native-like trimers imposes steric constraints on the host's glycosylation enzymes, resulting in an increase in the amount of oligomannose-type glycans at sites near the protomer interfaces (red/orange), compared to that of monomeric or non-native trimeric Env [168]. (b) Irrespective of the mature Env peptide sequence, the presence of a signal peptide (SP) from a transmitted/founder (TF) viral isolate results in an increase in oligomannose-type glycosylation, while a chronic-stage signal peptide results in increased complex-type glycosylation. The signal peptide influences Env trafficking, folding and retention through the ER [175]. (c) Modeling the ER α-mannosidase I (cyan, PDB: 5KIJ) on to its substrate glycan (green) reveals extensive clashes with neighboring glycans (red) sufficient to explain the formation of the intrinsic mannose patch [171]. (d) It is hypothesized that membrane-bound Env constructs display elevated glycosylation processing as they exhibit a different topology relative to the membrane-bound enzymes compared to soluble constructs, which are released into the lumen of the ER [173], [174]. Schematic of a membrane-bound glycan processing enzyme, based on the structure of a sialyltransferase (PDB: 6APL). (e) While the processing of many Env glycans is limited by protein-directed steric constraints, the fate of others is dependent on the glycosylation enzymes possessed by the host cell. The gp41 from BG505 SOSIP.664 expressed in Chinese hamster ovary (CHO) cells displays increased sialylation compared to the same protein expressed in human embryonic kidney (HEK) cells [94].

We have previously published a quantitative, site-specific glycan analysis of the recombinant BG505 SOSIP.664 trimers [38]. This revealed that the oligomannose signature observed on Env was largely accounted for by several PNGS containing solely under-processed, oligomannose-type glycans, usually dominated by Man_9_GlcNAc_2_. The remaining sites contained either processed, complex-type glycans, or a mixed population. Mapping this information onto the structure of BG505 SOSIP.664 illustrated how large regions of oligomannose-type glycans span across the outer domain of gp120, while complex-type glycans occupied sites at the periphery of the trimer, particularly on the gp41 subunits (Fig. 3).

The Paulson laboratory employed a complementary site-specific workflow to analyze BG505 SOSIP.664 trimers [163]. By sequential digestion of glycopeptides with Endoglycosidase H (to cleave oligomannose-/hybrid-type glycans, leaving a GlcNAc residue) and Peptide-*N*-Glycosidase F in the presence of ^18^O-water (to cleave the remaining complex-type glycans and convert the Asn to an ^18^O-labeled Asp), they were able to generate novel mass signatures for each category of glycan: + 203 Da for oligomannose-/hybrid-type, + 3 Da for complex-type glycans, and + 0 for unoccupied peptides. Their data were in very close agreement to our earlier analysis. This classification method is very powerful for low abundance samples (as heterogeneous glycopeptides become grouped as a single peptide), although information on the exact composition of glycan sites is not captured.

In some instances, the detail obtained from intact glycopeptide analysis may explain < 100% neutralization plateaus observed by some bnAbs [176], [177]. For instance, PGT135 is only able to neutralize around 85% of BaL pseudoviruses [176]. This bnAb recognizes predominantly glycans at N332, N386, and N392 (Fig. 2) [137]. Site-specific analysis of the N392 PNGS on recombinant gp120_BaL_ (subscript denotes strain) revealed the majority of glycans at this site to be Man_8_GlcNAc_2_ structures, with a secondary population of Man_9_GlcNAc_2_ [176]. In line with this, a crystal structure of the PGT135 Fab bound to a gp120_JR-FL_ core shows the N392 site to be occupied by a Man_8_GlcNAc_2_ structure [137]. Modeling an additional mannose residue to this glycan (to give Man_9_GlcNAc_2_) revealed steric clashes with the bnAb [137]. Furthermore, it has been shown that PGT135 is unable to neutralize pseudovirus displaying predominantly Man_9_GlcNAc_2_ structures [137]. Thus, Man_9_GlcNAc_2_ at the N392 site seems unoptimal for PGT135 binding, and the site-specific presence of this structure may account for the observed neutralization plateaus.

### Immunogen mimicry of viral glycosylation

Recent progress in the production and purification of HIV-1 virions has enabled the glycosylation analysis of virally derived Env. The Dell laboratory presented a qualitative analysis of gp120_BaL_ glycosylation derived from virions produced in a human lymphoid cell line [178]. In line with analysis of SOSIP.664 trimers, 15 of the 24 PNGS contained solely oligomannose-type structures, although the relative abundances of each glycoform were not determined. The remaining nine contained either complex-type structures or mixed populations [178].

We have since performed site-specific glycan analysis on gp120_BG505_ isolated from virions expressed in a similar lymphoid cell line [173]. This allowed for a comparison with BG505 SOSIP.664 trimers expressed in both human embryonic kidney (HEK) cells (as per previous analyses), and under equivalent conditions to current Good Manufacturing Practices (cGMP) in a Chinese hamster ovary (CHO) cell line. The analysis revealed that sites occupied by exclusively oligomannose-type glycans on virally derived Env were largely conserved on recombinant SOSIP.664s. This is reassuring considering a large proportion of glycan-targeting bnAbs recognize the oligomannose-type glycans at these sites (Fig. 2). However, there were some key differences between virally derived and recombinant Envs. Namely, virally derived Envs displayed increased levels of glycosylation processing, both in terms of the relative amount of complex-type glycosylation and in terms of the structures present (i.e., complex-type glycans on virally derived Envs were more branched). This discrepancy was generally attributed to several complex-type PNGS on virally derived Env that contained mixed populations of glycans on the SOSIP.664 trimers. We propose that this is due to the membrane-bound nature of virally derived Envs and will discuss this further in the Membrane tethering section.

The Paulson laboratory has also compared the glycosylation of virally derived Env from three strains (JR-FL, BG505, and B41), produced in peripheral blood mononuclear cells (PBMCs), with their corresponding SOSIP.664 trimers, produced in HEK cells, using their aforementioned site-specific classification method [174]. Their results were in general agreement with our observations that mixed sites on SOSIP.664 trimers tended to be fully processed on the virally derived Env. Thus, while there is some differential processing between viral and recombinant Env, many key bnAb epitopes are conserved. In terms of immunogen design, it is currently unknown whether absolute mimicry of viral glycosylation is required. This is likely to depend on the specific epitope targeted, for example, if the epitope mainly comprises the conserved base of the glycan.

### Glycan clustering and the intrinsic mannose patch

**Fig. 4:**
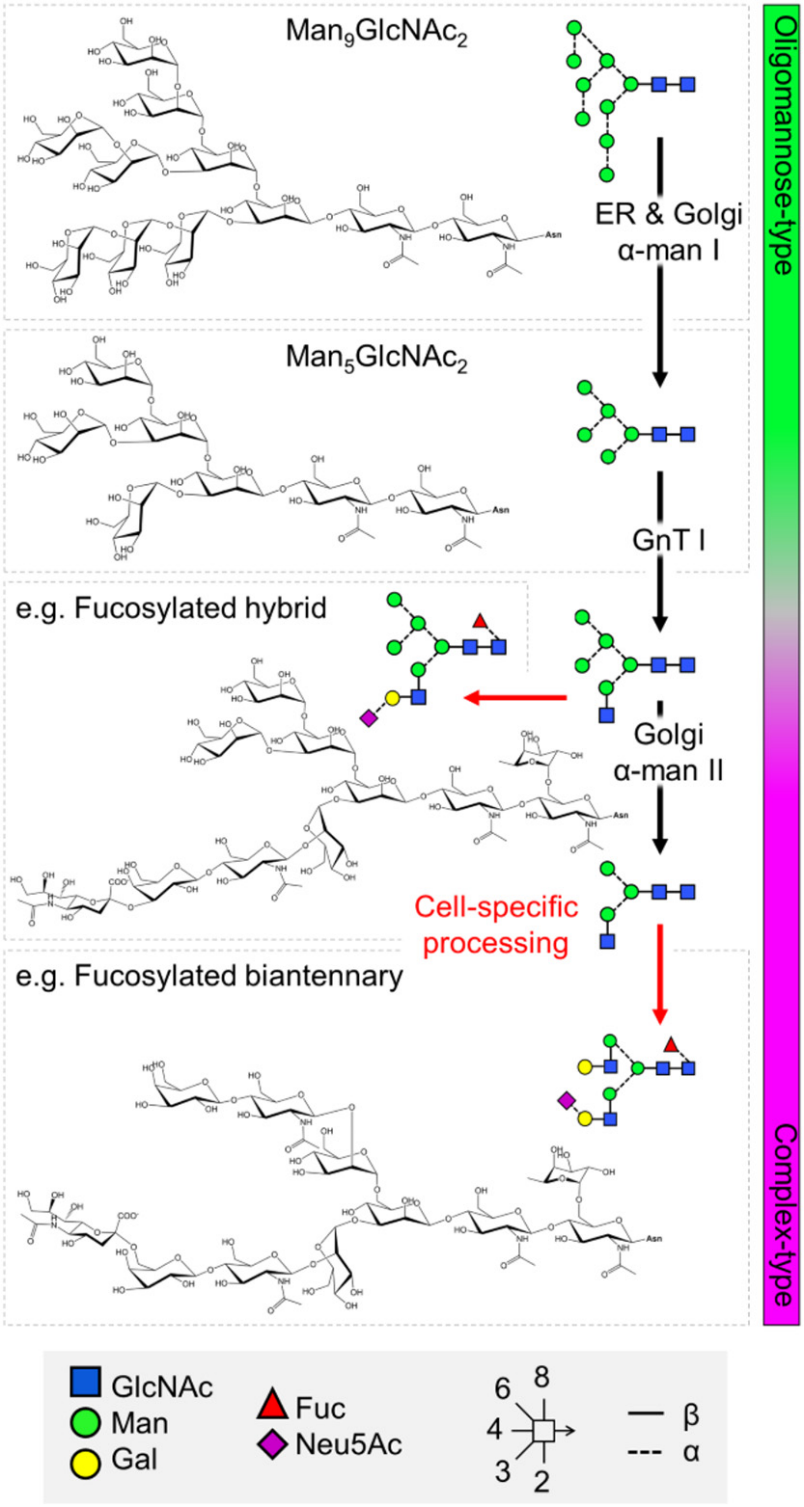
Overview of the mammalian *N*-glycosylation pathway. The envelope spike is extensively glycosylated by the host cell, which typically follows a highly ordered pathway. As the protein is translated, a Glc_3_Man_9_GlcNAc_2_ (Glc, glucose, Man, mannose, GlcNAc, *N*-acetylglucosamine) precursor is transferred en bloc to Asn residues within the *N*-glycan consensus sequence Asn-X-Thr/Ser (where X is any amino acid except Pro). As the protein is folded the three terminal glucose residues are removed to give rise to a glycoprotein displaying homogenous Man_9_GlcNAc_2_ structures. This is then further trimmed by endoplasmic reticulum (ER)- and Golgi apparatus-resident α-mannosidases to give rise to Man_5_GlcNAc_2_. Steric constraints within Env limit the actions of these early enzymes resulting in a population of under-processed oligomannose-type glycans. The addition of a β1-2-linked GlcNAc residue to Man_5_GlcNAc_2_ structures initiates cell-specific diversification to a variety of hybrid- and complex-type structures, through additional processing and/or trimming. α-man I and II, α-mannosidase I and II; GnT I, *N*-acetylglucosaminyltransferase I; Gal, galactose; Fuc, fucose; Neu5Ac, *N*-acetylneuraminic acid (sialic acid). Glycan structures are depicted in symbols according to the Consortium for Functional Glycomics nomenclature, with linkage information according to Oxford nomenclature, as shown in the key.

This abundance of oligomannose-type glycans on Env is somewhat unusual given the virus derives its glycan shield from the host cell glycosylation machinery. This typically follows a highly ordered pathway whereby oligomannose-type precursors are trimmed and rebuilt as complex- and/or hybrid-type glycans (Fig. 4). Two hypotheses existed as to why Env glycosylation diverges from that typically observed on mammalian glycoproteins. Either a proportion of Env glycoproteins are exiting the glycosylation pathway before encountering the later enzymes, or steric constraints exist within Env that are preventing complete processing by the earlier enzymes, or a combination of the two. Several pieces of evidence support the model of steric hindrance.

First, a proportion of oligomannose-type glycans are resistant to endoplasmic reticulum (ER) α-mannosidase I (the enzyme responsible for trimming Man_9_GlcNAc_2_ to Man_8_GlcNAc_2_, Fig. 4) digestion *in vitro* [159]. Expressing gp120 with kifunensine (an α-mannosidase I inhibitor) generates a glycoprotein bearing almost exclusively Man_9_GlcNAc_2_ structures, replicating the immature glycoprotein found in the early ER. Kinetic analysis of the hydrolysis of Man_9_GlcNAc_2_ to Man_8_GlcNAc_2_ revealed approximately half of the Man_9_GlcNAc_2_ to be rapidly trimmed to Man_8_GlcNAc_2_, with the remaining Man_9_GlcNAc_2_ processed at a much slower rate. However, there remained a proportion of Man_9_GlcNAc_2_ (~ 30%) that could not be hydrolyzed, even after exhaustive digestion. The resistance of these glycans to digestion *in vitro* supports the model of steric hindrance, likely caused by the high density of glycans on gp120 preventing α-mannosidases accessing their substrate glycans.

Each gp120 subunit contains between 18 and 33 (median, 25) PNGS [39]. To elucidate whether glycan density is the limiting factor preventing complete processing of the glycan shield, the number of PNGS on the outer domain of gp120 was correlated with the abundance of oligomannose-type glycans on sequences isolated from an individual over the course of infection, and on a cross-clade panel of 29 strains [157]. In both instances, a strong correlation was observed. This correlation was not seen when comparing the abundance of oligomannose-type glycans with the total number of PNGS on gp120 for the cross-clade panel (though it was for the infected patient sequences), suggesting that the incomplete processing observed on gp120 is driven by local glycan density, rather than overall glycan number. Similarly, Stewart-Jones *et al*. [149] observed the number of glycan processing steps to be significantly lower for “crowded” glycans (those with more than 15 PNGS within a 50-Å radius) than for “dispersed” glycans (those with fewer than 15 PNGS within a 50-Å radius).

The extent to which individual glycans contribute to the steric hindrance of glycosylation enzymes was investigated by the systematic site-directed mutagenesis of all the PNGS on gp120_BaL_, by mutating each Asn within the glycosylation sequon to Ala [156]. Although the removal of individual sites did not have a severe effect on the abundance of oligomannose-type glycans, several site deletions resulted in a larger than expected loss of Man_9_GlcNAc_2_, often accompanied by a compensatory increase in the lower oligomannose species, Man_5-8_GlcNAc_2_. The glycan sites accounting for the largest decrease in Man_9_GlcNAc_2_ generally mapped to the outer domain of gp120, further supporting a model whereby localised glycan clustering is sterically hindering early glycosylation processing. Thus, the loss of a PNGS within such clusters would increase the accessibility of neighboring glycans so that multiple nearby glycans exhibit increased processing. In further support of this hypothesis, it has recently been confirmed by site-specific glycan analysis that the disruption in glycan processing upon the loss of a PNGS is largely limited to those sites adjacent to the missing glycans [179].

**Fig. 5:**
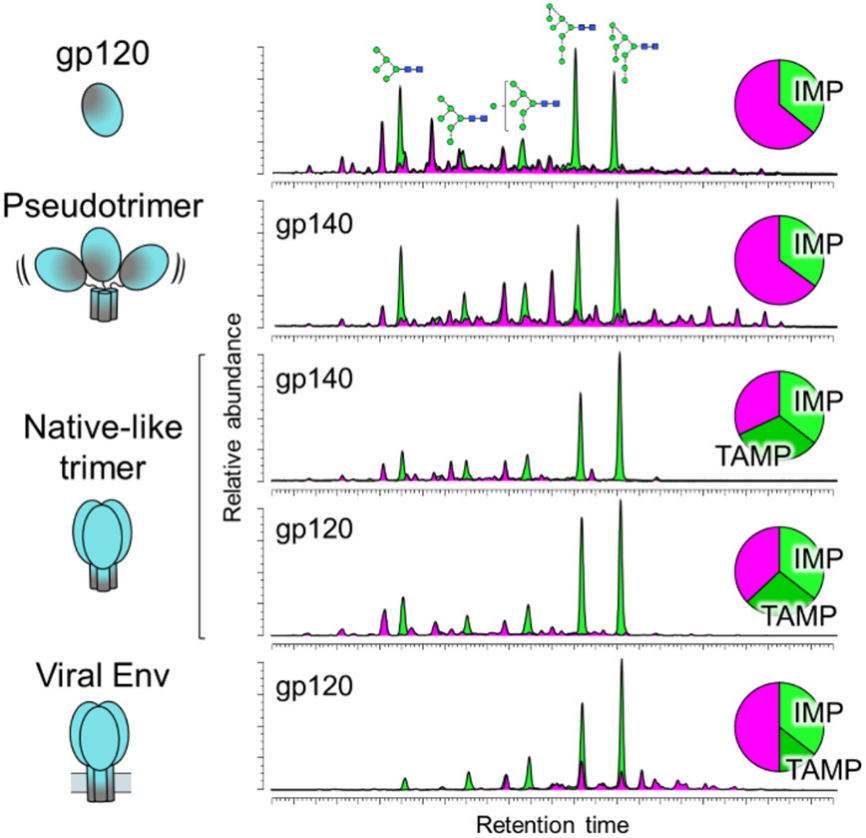
Glycan clustering and protein structure limit glycosylation processing on HIV-1. Quantitative glycan analysis of monomeric gp120, pseudotrimers, native-like trimers, and virally derived Env. For comparison, data for both gp120 and gp140 (gp120 + truncated gp41) from native-like trimers have been included, only data for virally derived gp120 were available [168], [173]. Each construct is based on the BG505 sequence containing the T332N mutation. Glycans were enzymatically released, fluorescently labeled, and analyzed by hydrophilic interaction liquid chromatography–ultraperformance liquid chromatography. Oligomannose-type glycans were quantified by their susceptibility to digestion with Endoglycosidase H. The chromatograms reveal a population of oligomannose-type glycans (green) intrinsic to all Env constructs, termed the intrinsic mannose patch (IMP). Only native-like trimers and virally derived Envs display the additional trimer-associated mannose patch (TAMP) signature, attributed to additional steric protection from processing.

Thus, while the glycan structures present on most glycoproteins are determined by the host cell, HIV-1 is able to partly modulate its glycosylation processing through the number and position of PNGS, as determined by the viral sequence. The number of PNGS on Env is conserved both across clades and longitudinally throughout infection [157], [158]. The resulting high glycan density on Env (and gp120 in particular) limits the actions of α-mannosidases and results in a large population of oligomannose-type glycans. This principle is illustrated in a model of the ER α-mannosidase I enzyme binding a substrate Man_9_GlcNAc_2_ glycan on the outer domain of gp120, which reveals extensive clashes with the surrounding glycans [171]. A similar phenomenon has been observed on the glycoprotein complex of Lassa virus, which also exhibits high localised glycan density resulting in under processed oligomannose-type clusters [180]. The oligomannose-type glycans of gp120 are therefore an inherent feature of the glycoprotein, known as the “intrinsic mannose patch” (IMP), and to some extent, all Env constructs will display this glycan signature (Fig. 5). Encouragingly, many of the aforementioned glycan-targeting bnAbs recognize the oligomannose-type glycans of the IMP. The overall resilience of the oligomannose population to sequence variation, and its presence on both immunogens and viral Env, supports this feature as a conserved target for vaccine design.

### Protein structure and the trimer-associated mannose patch

As the focus of immunogen design shifted from monomeric gp120 toward trimeric Env, so did the requirement to categorize glycans from native-like immunogens. It had been observed that trimeric Envs often displayed even higher levels of under-processed oligomannose-type glycosylation [94], [158], [159], [168]. The discrepancy in the abundance of oligomannose-type structures between monomeric and trimeric Env suggests that these structures are derived from an additional mechanism to the one driving the formation of the IMP. It was hypothesized that the “trimer-associated mannose patch” (TAMP) arose due to glycan‐glycan and glycan‐protein interactions at the protomer interface further sterically restricting processing enzymes [45], [158], [159].

Quantitative glycan analysis soon revealed that the TAMP glycan signature was only present on native-like trimers, with non-native pseudotrimers often displaying glycosylation patterns similar to that of monomeric gp120 (Fig. 5) [92], [94], [168]. Negative-stain EM revealed that non-native pseudotrimers often form open, irregular structures, in stark contrast to the well-ordered, propeller-shaped format of native-like trimers [92], [94], [171]. This structural difference is sufficient to explain the discrepancy in glycosylation, as open, irregular structures lack the additional steric protection of native-like trimers and allow for more complete glycan processing. Thus, although most Env proteins have a population of oligomannose-type glycans atypical of mammalian glycosylation, only native-like trimers display the additional TAMP signature. As such, glycosylation profiling is becoming a widely adopted tool that can be used to readily distinguish between native-like and misfolded trimers and guide the selection of immunogens [181].

Site-specific glycan analysis was applied to sequence-matched gp120_BG505_, uncleaved pseudotrimers, and native-like, BG505 SOSIP.664 trimers. The results revealed for the first time the individual PNGS that contribute to the TAMP. As hypothesized, sites located at the protomer interfaces on the native-like BG505 SOSIP.664 trimers displayed restricted glycan processing compared to both the pseudotrimers and monomeric gp120 (Fig. 3a). This was most significant at sites N156, N160, N197, and N276, which had > 40 percentage point increase in oligomannose-type glycans on BG505 SOSIP.664 compared to gp120 [168].

Crucially, in support of the native-like configuration of SOSIP.664 trimers, the TAMP signature is present on both virally derived Envs and native-like trimers (Fig. 5), although it is somewhat smaller on viral Env [173]. This is likely due to “TAMP sites” N197 and N276 displaying predominantly complex-type glycosylation on viral Env [173], [174]. The N156 and N160 “TAMP sites” are, however, conserved oligomannose-type sites in most instances [173], [174]. The N160 site has been reported as a complex-type site in JR-FL virions, highlighting potential strain-specific differences [174].

### Cell-specific glycosylation

While the abundant population of oligomannose-type glycans observed on Env is attributed to steric constraints imposed by the protein itself, the more accessible regions of the glycoprotein are subject to processing by the host cell's glycosylation pathway. Such processing is cell type-specific, dependent on the repertoire of glycosidases and glycosyltransferases expressed by the host cell [182].

A comparative analysis of BG505 SOSIP.664 derived from CHO cells and HEK cells illustrates the nature of protein- and cell-directed Envelope glycosylation [94]. Both CHO and HEK-derived material display similar levels of protein-directed glycosylation: oligomannose-type glycans account for 55% and 56% of the total glycan pool, respectively, with individual oligomannose species exhibiting similar distributions [94]. In contrast, the remaining complex-type glycans present considerable cell-specific differences. This is particularly evident on the gp41 subunit, with CHO-derived material containing substantially more sialylated structures than HEK-derived BG505 SOSIP.664 (Fig. 3e) [94]. The abundance of sialic acid is known to impact the antigenicity and immunogenicity of Env. Kong *et al*. [150] observed that gp120 expressed in an insect cell line modified to impart asialylated mammalian-type glycans was significantly more immunogenic than sialylated gp120 expressed in HEK cells.

A comparison of virally derived gp120 produced in PBMCs and gp120 from pseudovirus produced in HEK cells revealed similar cell-specific differences [183]. Namely, sialylated glycans from material produced in PBMCs displayed primarily α2-6-linkages, whereas HEK-derived material displayed only α2–3-linked structures [183]. It can be noted that α2–6-linked sialylated glycans have previously been associated with anti-inflammatory effects; thus, there may be potential immunological consequences of these structures [184].

Like oligomannose-type glycans, complex-type glycans have emerged as important in regard to the formation of bnAb epitopes. Some bnAbs, such as PGT121 display promiscuous recognition of both oligomannose- and complex-type glycans in glycan arrays [185]. Antibodies such as PGT151 are dependent on tri- and tetra-antennary structures at the gp120–gp41 interface [186], while α2-6-linked sialylated hybrid- and complex-type glycans have been implicated in the development and binding of apex-targeting bnAbs such as PG16 and the CAP256–VRC26 lineage [187], [188], [189]. Interactions between the sialic acid residues of Env and Siglecs (sialic acid-binding immunoglobulin-like lectins) have also been observed to play an important role in the infection of macrophages, which express low levels of cell-surface CD4 [190]. Thus, cell-directed glycosylation can play a role in both viral infectivity and bnAb binding, and understanding the factors influencing complex-type glycosylation may have implications in guiding immunogen design, for instance, when choosing expression cell lines.

### Membrane tethering

The majority of glycan analyses have been performed on soluble Env constructs due to the difficulties associated with expressing and purifying full-length, membrane-bound Env. Until recently, the impact of membrane tethering on Env glycosylation had remained largely unaddressed. Analyses of virally derived Envs, while performed on membrane-bound material, generally did not control for expression cell-line, known to influence glycosylation processing [173], [174]. However, recent studies by Rantalainen *et al*. and Cao *et al*. [174], [191]. directly compare the glycosylation of membrane-bound Envs with their corresponding soluble SOSIP.664 trimers expressed in the same cell lines. The differences observed between membrane-bound Env and SOSIP.664 are reminiscent of the differences observed between viral Env and SOSIP.664, in that sites containing mixed populations of glycans on the soluble SOSIP.664 trimer typically displayed only fully processed, complex-type glycan structures on the full-length construct [191]. It was hypothesized that the more complete processing observed on full-length constructs is due to membrane-bound Env being kept in closer proximity to the membrane-bound glycosylation enzymes throughout the ER and Golgi (Fig. 3d), as opposed to soluble Env which is released into the lumen [173], [174]. In line with this hypothesis, membrane-bound CD59 also displays increased processing compared to its soluble counterpart [192].

Alternatively, it has been hypothesized that the closer proximity of the membrane to full-length constructs, particularly to gp41, would pose additional steric constraints on Env glycosylation processing [171]. In support of this hypothesis, Panico *et al*. [178] observed exclusively oligomannose-type glycans at the membrane-proximal N88 site on virally derived Env. A caveat of all the above studies is that full-length constructs do not contain the SOSIP.664 stabilizing mutations, which may influence glycosylation via a separate mechanism, for example, the speed at which Env transits through the ER.

### Signal peptide

Like many membrane proteins, immature Env contains a signal peptide at the N terminus responsible for directing the nascent peptide to the ER, which is subsequently cleaved off prior to transport of the maturing peptide through the ER and Golgi (Fig. 3b). The signal peptide strongly influences the processing of Env as it transits through the ER, impacting factors such as trafficking to the ER, the rate of signal peptide cleavage, and the retention time of Env within the ER [193], [194], [195], [196], [197], [198]. Natural variation exists within the signal peptides of Env. For example, TF viral isolates often over represent His at position 12 of the signal peptide, a signature not usually observed in chronic phase viral isolates [198]. Given the influence of the signal peptide on the molecular biology of Env, the laboratory of Fauci sought to determine the impact of the natural variation observed between the signal peptides isolated from TF viruses and from a chronically infected patient on the glycosylation, structure, and antigenicity of Env. By creating four constructs containing either the gp120 sequence isolated from a TF or chronic virus, in combination with either their natural signal peptide, or that of the other virus (Fig. 3b), Yolitz *et al*. [175] assessed glycosylation and antigenicity by binding to various lectins and bnAbs. They found that despite gp120 sequences encoding for the same mature protein, the presence of a signal peptide from the TF viral isolate resulted in an increase in oligomannose-type glycosylation (as judged by binding to the *Narcissus pseudonarcissus* lectin and bnAb 2G12, specific to α-linked mannose residues), while the presence of a signal peptide from chronic-stage virus increased the amount of complex-type glycosylation (as judged by binding to *Ricinus communis* lectin, which preferentially binds structures terminating in galactose).

The results of Yolitz *et al*. are in line with the glycan analyses of Go *et al*. [165], who observed higher levels of oligomannose-type glycosylation on TF viruses than those isolated from chronically infected patients. However, a similar comparison of SOSIP.664 trimers isolated from early and late time points by the Paulson laboratory showed very similar glycan processing [191]. Nevertheless, the findings of Yolitz *et al*. implicate the signal peptide, a domain that does not appear in the mature protein, as a regulator of glycosylation processing.

### Occupancy

Potential *N*-glycosylation site occupancy has recently emerged as an important aspect of Env glycosylation. The Paulson laboratory employed site-specific glycan classification analysis to BG505 SOSIP.664 trimers to assess PNGS occupancy [163]. They found that overall occupancy was very high: all but four of the 28 PNGS (V1/V2 and gp41 sites N190, N197, N618, and N625) were > 90% occupied and none were < 50% occupied [163]. Extension of their method to native-like trimers derived from different strains (JR-FL, B41, CRF02_AG_250, 327c, PC64) confirmed that the V1/V2 and gp41 sites were most susceptible to under-occupancy [174], [191]. The reports concerning occupancy of gp41 sites, particularly N625, seem to vary, perhaps due to methodological or sample variation. For example, Guttman *et al*. [199] have reported occupancy < 20% at this site in BG505 and KNH1144 SOSIP.664 trimers, which is also compatible with the known epitope of the 35O22 bnAb [82].

Interestingly, virally derived and membrane-bound Env trimers are generally more occupied than their SOSIP.664 counterparts [173], [174], [191]. Although the mechanism through which PNGS under-occupancy arises is not yet understood, we hypothesize that the codon-optimization process, used to increase the yield of recombinant SOSIP.664 trimers, may increase the rate of protein translation and folding and reduce the chance of an *N*-glycan being attached [173]. This may be particularly true of glycans in the V1/V2 region where several PNGS are in close proximity.

### Considerations for immunogen manufacture

All of the above factors influence the glycosylation processing of Env to varying degrees. Although the extent to which Env immunogens must mimic the native viral envelope spike is not yet known, there are nevertheless important considerations for immunogen design and manufacture. In particular, expression cell-type and PNGS under-occupancy may yet emerge as crucial aspects in the cGMP production of Env immunogens, as under-occupancy may introduce unfavourable, immunodominant autologous nAb epitopes. This is particularly relevant in light of Wagh *et al*. [76], observing increased neutralization breadth in individuals infected with TF viruses with more intact glycan shields.

## Understanding the Nature of Glycan-Dependent bnAb Epitopes

The structural characterization of multiple bnAbs in complex with Env by both x-ray crystallography and EM has enabled a detailed description of the epitopes of many bnAb (Table 1) [202], [213]. Furthermore, it has revealed many of the unusual antibody features required for broad neutralization. Nevertheless, there remained many unknowns about the precise nature of glycan epitopes. This is, in part, due to difficulties in resolving extremely heterogeneous glycans with structural techniques that rely on the averaging of many molecules to reveal the consensus structure [122]. Glycan arrays are also a useful tool in assessing the glycan-binding properties of bnAbs, although these too have limitations (e.g., if the epitope comprises protein components). Site-specific glycan analysis has gone some way to bridging these knowledge gaps. While there is generally good agreement of the precise structures occupying glycan epitopes between glycan analyses and structural studies/arrays, a few discrepancies remain. For example, there are several reports on the preference of apex-targeting bnAbs PG9 and PG16 for complex- or hybrid-type glycans (specifically α2-6-linked sialylated structures) at the N156 site [187], [188], [208]. However, these structures are seldom seen at this site in glycan analyses of recombinant, soluble trimers or virally derived Env [38], [163], [173], [174].

**Table 1:** Examples of glycan-targeting broadly neutralizing antibodies and their glycan epitopes

Epitope	bnAb	PNGS	Glycan type	Refs.
Outer domain glycans	PGT130	N301, N332/N334	Oligomannose	[200], [201]
	2G12	N295, N332, N339, N392	Oligomannose, specifically α1-2-linked motifs on Man_8-9_GlcNAc_2_ structures	[124], [125], [126], [127], [128], [129], [130]
	VRC-PG05	N262, N295, N448	Oligomannose	[123]
	PGT135	N295, N332, N386, N392	Oligomannose	[137], [176], [201]
	PGT128	N156, N295, N301, N332/N334	Oligomannose	[37], [136], [200], [201]
	PGT122	N137, N156, N301, N332	Oligomannose	[149], [201], [202]
	PGT121	N137, N156, N301, N332/N334	Oligomannose or complex	[142], [185], [200], [201]
	BG18	N156, N332, N386, N392	Oligomannose, possibly complex at N156	[203]
	10-1074	N156, N301, N332	Oligomannose, possibly complex at N156	[185], [204]
V1/V2 glycans	PGT145	N160	Oligomannose	[134], [201]
	CH04	N160	Kifunensine abrogates neutralization (cannot tolerate Man_9_GlcNAc_2_)	[205]
	PGDM1400	N160	Kifunensine reduces/abrogates neutralization (cannot tolerate Man_9_GlcNAc_2_)	[206]
	VRC38.01	N133, N156, N160	*N*-acetylglucosamine core of N133	[207]
	PG9 and PG16	N173 (N156), N160	Oligomannose at N160, hybrid or bi-antennary structures at N156/N173, specifically containing α2-6-linked terminal sialic acids	[187], [188], [208], [209], [210], [211]
	CAP256–VRC26 (lineage)	N156, N160	Oligomannose at N160, hybrid at N156, or bi-, tri-, tetra-antennary structures containing α2-6-linked terminal sialic acids	[133], [189]
	PCT64 (lineage)	N156, N160	Oligomannose, preferentially Man_5_GlcNAc_2_	[212]
CD4bs proximal	3BNC117	N197, N276	*N*-acetylglucosamine cores	[134], [149]
	PGV04 (VRC-PG04)	N276, N301, N363, N386	Enzymatic de-glycosylation does not affect binding (no strong glycan-dependence)	[213], [214]
	HJ16	N276	*N*-acetylglucosamine core	[215], [216], [217]
	IOMA	N197, N276, N363	Complex, minor contact with oligomannose glycan at N363	[204]
	179NC75	N276	Oligomannose or hybrid^a^	[218]
	VRC01	N276	*N*-acetylglucosamine core	[131], [149]
gp120-gp41, interface	8ANC195	N234, N276, N637	Kifunensine did not affect neutralization (can tolerate Man_9_GlcNAc_2_)	[219], [220]
	PGT151	N611, N637	Complex, specifically tri- and tetra-antennary structures	[186], [221], [222]
	VRC34.01	N88	Kifunensine, swainsonine or GnTI^−/−^ cells^b^ minimally affect neutralization (not dependent on complex structures)	[223]
	ACS202	N88	Kifunensine, or GnTI^−/−^ cells minimally affect neutralization (not dependent on complex structures)	[224]
	35O22	N88, N230, N241	Oligomannose	[149], [225]

a In Freund *et al*. [218] the loss of binding of 179NC75 following expression of BG505 SOSIP.664 trimers with kifunensine was used to argue a dependency on complex-type glycans. However, they also report a loss of binding to gp120 after digestion with Endoglycosidase H (which cleaves oligomannose- and hybrid-type glycans). This suggests that 179NC75 recognizes Man_8-5_GlcNAc_2_ or hybrid-type structures. In line with this, site-specific glycan analysis of BG505 SOSIP.664 trimers reports predominantly Man_8-5_GlcNAc_2_ glycans at the N276 site, with a small population of hybrid-type structures [38].

b Treatment with swainsonine (α-mannosidase II inhibitor) results in oligomannose- and hybrid-type glycosylation. Expression in GnTI^−/−^ cells (deficient in *N*-acetylglucosaminyltransferase I) results in Man_5-9_GlcNAc_2_ structures only.

## Beyond Antigenic Mimicry

Native-like trimers are excellent structural mimics of viral Env, display similar glycosylation profiles, and are capable of binding bnAbs. Yet immunogenicity studies with native-like trimers have only routinely elicited autologous Tier-2 nAbs and heterologous Tier-1 nAbs [107], [116], [117], [118], [119], [120], [121]. Weakly neutralizing heterologous Tier-2 antibodies have also been reported in rabbits [104]. With the exception of cows (whose antibodies naturally contain very long HCDR3), the potent bnAbs required for a protective vaccine have not yet been generated via immunization with native-like trimers [58]. The elicitation of bnAbs against the envelope spike will inevitably be challenging. Broadly neutralizing epitopes are broad due to their conserved nature, although conservation generally correlates with poor immunogenicity as otherwise the epitope would have been selected against by immune pressure. In this section, we will address some of the strategies aimed at increasing the immunogenicity of this target.

### Mimicry of glycan epitopes: chemical approaches

The first of many glycan-binding bnAbs to be discovered was 2G12. To date, this the only bnAb identified to exclusively bind glycans. The 2G12 antibody recognizes clusters of α1-2-linked mannose motifs present on the Man_8-9_GlcNAc_2_ structures of the IMP (Fig. 2) [124], [125], [126], [127], [128], [129], [130]. Glycan-based vaccine strategies have thus far centered on the design of immunogens that mimic the 2G12 epitope. Several groups have created immunogens displaying multivalent, chemically synthesised oligomannose-type glycans attached to various scaffolds, including the following: carbohydrate, cholic acid, cyclic peptide, dendrimer, DNA, and gold (reviewed by Wang [226]). Although many of these constructs are able to bind 2G12, and could elicit antibodies with specificity to oligomannose-type glycans, none have induced antibodies capable of neutralizing HIV-1. This may be due to the inability of these antibodies to recognize clusters of oligomannose-type glycans as they are presented on Env, perhaps due to their non-domain-exchanged structures. Domain-exchange (at least for 2G12) is a requirement for HIV-1 neutralization [227].

The mimicry of other glycan-targeting bnAb epitopes has been explored with the design of peptide-based immunogens. Glycopeptides based on the V1/V2 region, containing the N156 and N160 PNGS, are capable of binding both apex targeting bnAbs and their unmutated common ancestors [208], [228], [229]. Similarly, glycopeptides based on the V3 region, containing the N332 PNGS, bound outer domain targeting bnAbs such as PGT128 [230]. In immunogenicity studies, V3 glycopeptides elicited antibodies capable of binding gp120 but unable to neutralize virus [230], [231]. The failure of antigenic mimics of bnAb epitopes to induce bnAbs further highlights the complicated relationship between the antigenicity and immunogenicity of these epitopes.

### Mimicry of glycan epitopes: biological approaches

**Fig. 6:**
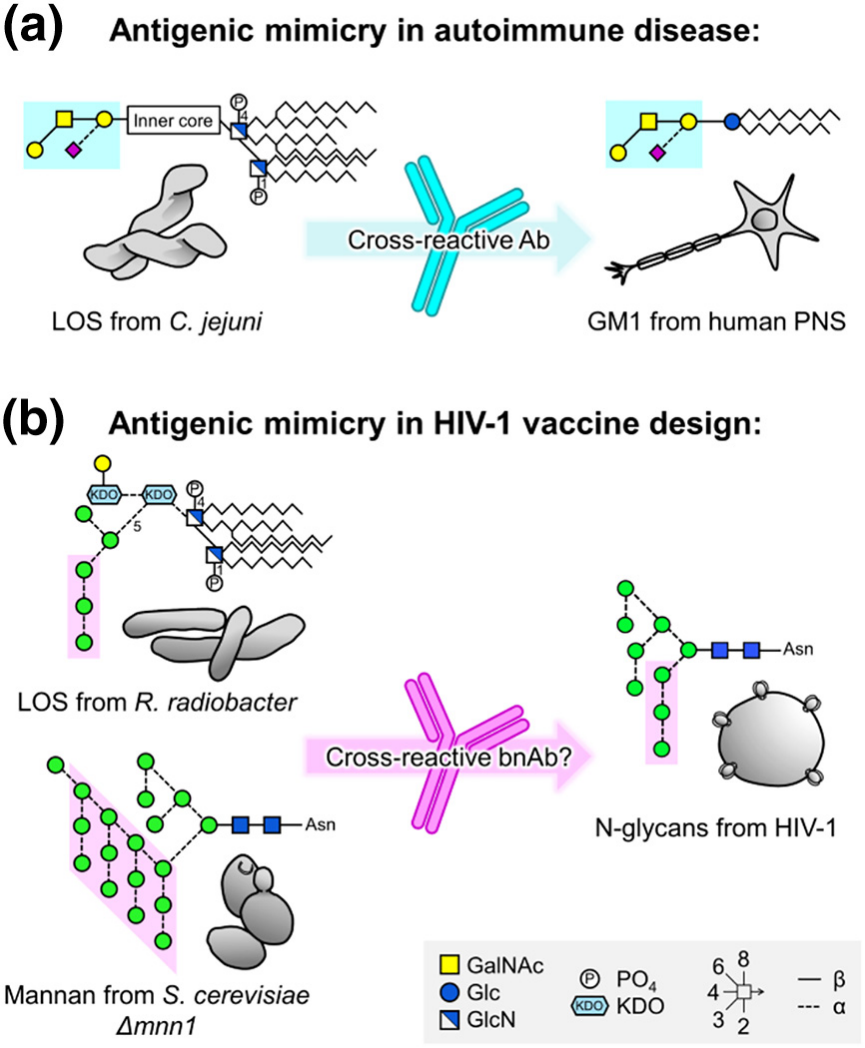
Antigenic mimicry in autoimmune disease and HIV-1 vaccine design. (a) Antigenic mimicry of lipooligosaccharides (LOS) from *C. jejuni* causes Guillain–Barré syndrome, an autoimmune response against the GM1 ganglioside in the peripheral nervous system (PNS). (b) LOS from *Rhizobium radiobacter* Rv3 and *Saccharomyces cerevisiae* deficient in the Mnn1 gene display glycan structures terminating in α1-2-linked mannose residues, mimicking the 2G12 epitope on HIV-1. GalNAc, *N*-acetylgalactosamine; Glc, gluscose; GlcN, glucosamine; KDO, 2-keto-3-deoxy-D-manno-octulosonic acid. Figure adapted from Scanlan *et al*. [46].

A potential failure of the above strategies may be that, while the oligomannose-type glycans of the HIV-1 glycan are not typically observed on mammalian glycoproteins, they are still fundamentally “self” structures. As noted above, this poses a challenge for the development of glycan-targeting bnAbs, as cross-reactive B cells are likely to be deemed autoreactive and will be negatively selected for. The fact that bnAbs generally do not arise until after several years of infection could support the notion that they are operating at the edge of immunological tolerance, or may simply reflect the long maturation process. However, tolerance to self-structures can be broken. For example, in some cases, infection with *C. jejuni* elicits an antibody response against its bacterial lipooligosaccharides that is able to bind nearly identical structures on the gangliosides of the peripheral nervous system (Fig. 6a) [148]. The resulting autoimmune disease, Guillain–Barré syndrome, provides proof of concept that immunological tolerance to “self” glycans can be broken by exposure to micro-organisms bearing similar structures [46].

It was hypothesized that 2G12 may have originally evolved to recognize high-mannose structures on pathogens other than HIV-1 [46]. Scanlan explored this further by assessing the reactivity of 2G12 to various yeast species [146]. Yeast typically display high-mannose structures that comprise an α1-6-linked mannnose backbone from which branch repetitive α1-2-linked mannose motifs (i.e., a key feature of the 2G12 epitope), sometimes capped with α1-3-linked mannose residues. Of the yeast species tested, 2G12 was able to bind to two, binding *Candida albicans* with similar affinities to that of gp120 [146], [232]. This interaction was glycan dependent as binding was inhibited by D-fructose, a known ligand for the 2G12 binding site. In addition, a non-glycan-dependent control bnAb was unable to bind any of the yeasts. Thus, the 2G12 antibody may equally well be described as an anti-*Candida* antibody with cross-reactivity to HIV-1 [146].

Scanlan went on to formally elucidate the relationship between the antigenicity and immunogenicity of yeast glycosylation [233]. *Saccharomyces cerevisiae* deficient in *mnn1*, the gene responsible for the α1-3-linked mannnose caps, displays mannans terminating in the α1-2-linked mannose motif (Fig. 6b). Immunization of rabbits with *S. cerevisiae ∆ mnn1* elicited antibodies with similar glycan specificities to 2G12. Furthermore, the sera displayed weakly neutralizing activity against HIV-1 [233]. The Doms laboratory also investigated using yeast as an HIV-1 immunogen. By knocking-out three genes they produced *S. cerevisiae* displaying predominantly Man_8_GlcNAc_2_ glycan structures. Although these yeasts now displayed a “self” glycan structure, immunization of rabbits elicited oligomannose-specific antibodies, capable of binding monomeric gp120, but unable to neutralize the virus [234]. As mentioned previously, an explanation for this apparent contradictory result may be that while the elicited antibodies were capable of recognizing isolated oligomannose-type glycans, they were not able to recognize densely packed clusters of oligomannose-type glycans as they are presented on Env. Thus, a logical next step may be to present immunogenic yeast glycan structures in the context of Env, for instance, by expressing Env in yeast. The resulting immunogens should display clusters of “non-self” glycans capable of breaking immunological tolerance to the clusters of oligomannose-type glycans present on Env.

A similar phenomenon has been observed by the Pantophlet laboratory. As per *S. cerevisiae ∆ mnn1* mannans, the lipooligosaccharides of *Rhizobium radiobacter* Rv3 bacteria contain a glycan motif analogous to the 2G12 epitope on HIV-1 (Fig. 6b) [235], [236]. Immunization of mice with *R. radiobacter* Rv3 also generated antibodies capable of weakly cross-reacting with HIV-1 [235], [237], representing another candidate micro-organism to investigate for a potential HIV-1 glycan-based vaccine.

### Mimicking bnAb development in natural infection

The development of bnAbs in a subset of infected individuals typically only occurs after a few years of infection. Neutralizing antibodies, however, arise early in infection and exert a considerable selection pressure on the virus. Repeated rounds of viral escape and antibody evolution ultimately drive the development of nAb breadth [70], [132], [133]. The discovery that native-like trimers can induce autologous Tier-2 nAbs could therefore be an important first step towards development of bnAbs. Accordingly, a line of research is to attempt to mimic the development of bnAbs in natural infection by immunizing with a longitudinal sequence of trimers based on the Envs from an infected individual who went on to generate bnAbs. An in-depth understanding of viral and antibody co-evolution throughout the course of natural infection will no doubt prove invaluable in aiding the design of such immunization regimens [132], [191], [212], [238], [239].

An alternative, albeit closely linked, approach is to design immunogens specifically targeted to initiate bnAb development. As noted previously, the development of bnAbs requires the activation of B cell lineages expressing gl-bnAbs, which are typically very poor at binding Env trimers [61], [62], [63], [64]. This approach therefore involves the modification of immunogens in order to better engage gl-bnAbs [240], usually by the deletion of PNGS and variable loops to remove the steric occlusions which prevent gl-bnAb binding [241], [242], [243]. The laboratories of Schief and Nussenzweig have reported that BG505 SOSIP trimers containing, among other mutations, N133 and N137 PNGS deletions, are able to bind a germline-reverted version of the outer-domain glycan-targeting bnAb, PGT121 [244]. Immunization of mice engineered to express the predicted germline of PGT121 with this trimer, prior to boosting with a sequence of immunogens containing decreasing modifications, was able to elicit heterologous Tier-2 neutralizing responses [245]. This provides proof of concept that immunization with specifically designed immunogens can initiate bnAb development. There is, however, an apparent conflict between the need to delete PNGS sites in order to initiate bnAb development, and the recent observation that bnAbs were more likely to develop in individuals infected with isolates containing more intact glycan shields [76].

## Conclusions

Antigenic mimicry is fundamental to most licensed vaccines. It is widely acknowledged that a protective HIV-1 vaccine will be based on the mimicry of the HIV-1 envelope spike, the sole target for bnAbs raised during infection. In addition to protein mimicry, Env-based immunogens will likely have to exhibit glycan mimicry, as many of the most potent bnAbs isolated to date recognize glycan structures within their epitopes. Thus, the characterization of glycan epitopes and an understanding of the principles governing their correct processing are needed in order to continue to guide the rational design of HIV-1 immunogens. Structural constraints imposed by the formation of native-like trimers restricts aspects of glycosylation processing, thus glycan analysis can help distinguish between native-like and non-native immunogens. However, native-like immunogens alone are not sufficient to induce bnAbs. As broadly neutralizing epitopes are inherently immunoquiescent, it is likely that additional strategies will be required in order to boost their immunogenicity.
